# Prioritizing Kidney Transplantation During COVID-19: A Single-Center Experience and Lessons for Future Surge Planning

**DOI:** 10.7759/cureus.95321

**Published:** 2025-10-24

**Authors:** Abishek Balakrishnan, Keir Mehta, Aydin Caglayan

**Affiliations:** 1 School of Medicine, Cardiff University, Cardiff, GBR; 2 School of Health Sciences, University of Manchester, Manchester, GBR; 3 General Surgery, Medway NHS Foundation Trust, London, GBR

**Keywords:** covid-19, kidney transplantation, ments, prioritization, renal transplant outcomes

## Abstract

Background

The COVID-19 pandemic disrupted elective surgical services, leading to the adoption of prioritization frameworks such as the Medically Necessary, Time-Sensitive (MeNTS) score. Cohort data from the initial pandemic year remains relevant when contextualized by later developments (vaccination rollout, targeted therapeutics, and recovery of transplant services). We evaluated case selection at the Cardiff Transplant Unit using MeNTS and early outcomes for patients considered for kidney transplantation during the first year of the pandemic.

Methodology

This single-center, retrospective, cohort study included adult patients considered for kidney transplantation between March 1, 2020, and March 1, 2021. MeNTS scores were calculated retrospectively for patients who received transplantation (n = 62) and those whose procedures were suspended (n = 45). Primary outcomes included early graft function and 90-day survival, one-year survival, and incidence/severity of COVID-19 infection.

Results

In total, 62 patients received transplantation, and 45 were suspended. The median cumulative MeNTS score was 58 (range = 52-64) in transplanted patients versus 61 (range = 57-65) in suspended patients (mean = 57.5 vs. 61.6; Mann-Whitney U = 401, Z = -6.269, p = 3.6 × 10^-10^). One-year patient survival was high in both groups (transplanted, 96.7% vs. suspended, 97.8%). Delayed graft function occurred in 20/62 transplanted patients (32%); 5/62 (8.1%) subsequently developed graft failure. Overall, 14 patients (13% overall; nine transplanted, five suspended) acquired COVID-19 infection during follow-up. Of these patients, nine required hospitalization, three required intensive care, and one patient died. The median three-month estimated glomerular filtration rate among transplanted patients was 53 mL/minute/1.73 m² (range = 16-90).

Conclusions

During the first pandemic year, the Cardiff Transplant Unit prioritized patients with lower MeNTS scores and observed acceptable short-term patient and graft outcomes. While MeNTS provided a transparent triage framework, transplant-specific adaptation and prospective validation are needed to guide prioritization in future system shocks.

## Introduction

The COVID-19 pandemic caused an abrupt and widespread disruption to elective surgical services, forcing many transplant programs to reduce activity or temporarily suspend services and reconfigure to manage surges in hospital admissions and preserve critical care capacity [1]. Early surveys and registry reports documented major reductions in organ procurement and transplantation, particularly affecting living-donor programs and elective kidney transplants [2].

In response, clinicians and health systems deployed prioritization frameworks to guide allocation of scarce operative capacity in a transparent, reproducible manner. The Medically Necessary, Time-Sensitive (MeNTS) scoring system, which integrates procedure, disease, and patient factors to weigh individual benefit against resource use and infection risk, became a widely referenced tool for surgical triage early in the pandemic and was rapidly adapted across specialties [3,4].

Kidney transplantation raised distinct ethical and clinical challenges for such triage. Recipients require lifelong immunosuppression that may increase susceptibility to severe COVID-19 infection and attenuate vaccine responses, yet transplantation confers substantial survival and quality of life benefits relative to remaining on dialysis [5]. Additionally, delaying transplantation imposes well-recognized harms from prolonged dialysis exposure and potential loss of organ offers [6]. These competing considerations complicate simple prioritization rules and motivate validation of transplant-specific adaptations to general triage tools.

The risk landscape evolved quickly after the first wave. Large-scale vaccination programs substantially altered population risk, but vaccine immunogenicity and effectiveness are attenuated in many solid organ transplant recipients, who show lower serological responses and reduced vaccine effectiveness compared with immunocompetent populations [7]. These findings have driven recommendations for enhanced primary and booster schedules and for prioritizing access to early outpatient antiviral therapy in high-risk groups.

Given these considerations, re-examining early prioritization decisions and outcomes in real-world transplant cohorts can inform refinements to triage tools and resilience planning for future system shocks. The primary objective of this study was to retrospectively evaluate the application of the MeNTS scoring system to kidney transplant candidates at the Cardiff Transplant Unit during the first pandemic year (March 1, 2020, to March 1, 2021). The secondary aims were to compare selection patterns between transplanted and suspended patients, describe early clinical outcomes (early graft function, delayed graft function (DGF), graft failure, and short-term survival), and consider implications for transplant-specific prioritization in future crises.

## Materials and methods

Study design and setting

We performed a retrospective cohort study of adult patients considered for kidney transplantation at the Cardiff Transplant Unit between March 1, 2020, and March 1, 2021. The study is reported in accordance with the STROBE statement. Local research governance and ethical approval were obtained from Cardiff and Vale University Health Board Research & Development.

Participants and eligibility

All consecutive adult patients (aged ≥ 18 years) who were either transplanted during the study window (transplanted group, n = 62) or had transplantation suspended without proceeding to surgery during the same period (suspended group, n = 45) were included. Pediatric recipients and planned multi-organ transplantations were excluded from the primary analysis; multi-organ cases are described separately where relevant.

Data sources and collection

Clinical data were extracted from the transplant database (Vitaldata) and electronic health records (Welsh Clinical Portal), supplemented by review of paper notes when necessary. Extracted variables included demographics (age, sex, ethnicity), comorbidities (diabetes, cardiovascular disease, respiratory disease), dialysis modality and duration, primary renal diagnosis, prior transplantation, donor type (living, donation after brain death (DBD), donation after circulatory death (DCD)), cold-ischemia time, immunosuppression regimen, perioperative complications, and length of stay. All data were pseudonymized at source and stored on secure hospital servers according to institutional governance policy.

MeNTS scoring

The MeNTS scoring system [3] was applied retrospectively to each case using documentation closest to the operative triage decision (within 30 days, where available). The MeNTS system comprises procedure, disease, and patient-related domains scored 1-5; higher scores indicate greater perioperative risk or resource use. Table 1 presents the modified rubric used for this study, adapted from the original publication. Two clinicians (AB, KM) independently assigned MeNTS scores for all cases while blinded to subsequent clinical outcomes (DGF, graft failure, death) and to documented COVID-19 infection status. Where scorers disagreed, a third senior clinician (AC) adjudicated the final score.

**Table 1:** MeNTS scoring tool (modified from Prachand et al. [3]). MeNTS: Medically Necessary, Time-Sensitive; OR: operating room; LOS: length of stay; ICU: intensive care unit; MIS: minimally invasive surgery; ONHS: otolaryngology head and neck surgery; GI: gastrointestinal; COPD: chronic obstructive pulmonary disease; CF: cystic fibrosis; CPAP: continuous positive airway pressure; HTN: hypertension; CHF: congestive heart failure; CAD: coronary artery disease; ILI: influenza-like illness

	Variable	1	2	3	4	5
Procedure factors	OR time, minutes	<30	31–60	61–120	121–180	>180
	Estimated LOS	Outpatient	<23 hours	24–48 hours	2–3 days	>4 days
	Postoperative ICU need, %	Very unlikely	<5	5–10	11–25	>25
	Anticipated blood loss, cc	<100	100–250	250–500	500–750	>750
	Surgical team size, n	1	2	3	4	>4
	Intubation probability, %	<1	1–5	6–10	11–25	>25
	Surgical site	None of the following row variables	Abdominopelvic MIS	Abdominopelvic open surgery, infraumbilical	Abdominopelvic open surgery, supraumbilical	OHNS/upper GI/thoracic
Disease factors	Nonoperative treatment option effectiveness	None available	Available, <40% as effective as surgery	Available, 40% to 60% as effective as surgery	Available, 61% to 95% as effective as surgery	Available, equally effective
	Nonoperative treatment option resource/exposure risk	Significantly worse/not applicable	Somewhat worse	Equivalent	Somewhat better	Significantly better
	Impact of a 2-week delay on disease outcome	Significantly worse	Worse	Moderately worse	Slightly worse	No worse
	Impact of a 2-week delay on surgical difficulty/ risk	Significantly worse	Worse	Moderately worse	Slightly worse	No worse
	Impact of a 6-week delay on disease outcome	Significantly worse	Worse	Moderately worse	Slightly worse	No worse
	Impact of a 6-week delay on surgical difficulty/ risk	Significantly worse	Worse	Moderately worse	Slightly worse	No worse
Patient factors	Age, years	<20	21–40	41–50	51–65	>65
	Lung disease (asthma, COPD, CF)	None	-	-	Minimal (rare inhaler)	>Minimal
	Obstructive sleep apnoea	Not present	-	-	Mild/moderate (no CPAP)	On CPAP
	CV disease (HTN, CHF, CAD)	None	Minimal (no meds)	Mild (1 med)	Moderate (2 meds)	Severe (!3 meds)
	Diabetes	None	-	Mild (no meds)	Moderate (PO meds only)	>Moderate (insulin)
	Immunocompromised	No			Moderate	Severe
	ILI symptoms (fever, cough, sore throat, body aches, diarrhoea)	None (asymptomatic)	-	-	-	Yes
	Exposure to a known COVID-19 positive person in the past 14 days	No	Probably not	Possibly	Probably	Yes

Outcomes

The primary outcomes were early graft function (estimated glomerular filtration rate (eGFR) at discharge and at three months, calculated using the CKD-EPI equation) and 90-day patient survival. Secondary outcomes included DGF (requirement for dialysis within seven days post-transplant), graft failure (return to long-term dialysis or re-transplantation), acute rejection (biopsy-proven or clinically treated), perioperative complications, length of hospital stay, and incidence and severity of laboratory-confirmed COVID-19 infection during follow-up. Follow-up began on the date of transplant for transplanted patients and on the date of suspension for suspended patients. Censoring occurred at death, graft failure, loss to follow-up, or the last clinical encounter.

Statistical analysis

Continuous variables were assessed for normality using the Shapiro-Wilk test and summarized as mean (SD) for normally distributed data or median (interquartile range) for skewed distributions. Categorical variables were summarized as counts and percentages. Between-group comparisons were performed using Student’s t-test (two-tailed) for normally distributed continuous variables; Mann-Whitney U test for non-parametric continuous variables (e.g., MeNTS scores); and chi-square test or Fisher’s exact test for categorical variables, depending on cell size. Associations between MeNTS score and binary outcomes (DGF, COVID-19 infection) were explored using logistic regression with odds ratios (ORs) and 95% confidence intervals (CIs). Correlation between MeNTS score and continuous outcomes (eGFR) was assessed using Spearman’s rank correlation coefficient (ρ). All tests were two-sided with a threshold for statistical significance set at p-values <0.05. All analyses were performed using R version 4.2.2 (R Foundation for Statistical Computing, Vienna, Austria).

## Results

Cohort and follow-up

Between March 1, 2020, and March 1, 2021, 107 adult patients were considered for kidney transplantation and met the inclusion criteria. Of these, 62 (57.9%) proceeded to transplantation, and 45 (42.1%) had transplantation suspended during the study window. We note the sample size imbalance between groups (62 vs. 45), which reduces comparative power and should be considered when interpreting between-group comparisons. Three deaths occurred during follow-up (two in the transplanted group, one in the suspended group).

Baseline characteristics

Baseline demographic and clinical characteristics are summarized in Table 2. Transplanted patients were younger than suspended patients (median age = 48.5 years, range = 18-74 vs. 64 years, range = 28-75). Two baseline characteristics differed significantly between groups, i.e., age (median = 48.5 vs. 64 years, p < 0.001) and dialysis modality (suspended patients were more likely to be on in-center hemodialysis; p < 0.001). Donor types among transplanted patients were 34 (54.8%) DBD, 16 (25.8%) DCD, and 12 (19.4%) living donors; seven (11.3%) transplanted patients underwent simultaneous kidney-pancreas transplantation. The distribution of sex did not differ significantly between groups (male sex: transplanted 41/62 (66.1%) vs. suspended 26/45 (57.8%); χ² = 0.79, p = 0.37).

**Table 2:** Baseline demographic and clinical characteristics of transplant and suspended patient cohorts. Data are presented as median (range) or number (%). P-values are from: ¹Comparison between transplanted and suspended groups; ²Mann-Whitney U test for continuous variables; ³χ² or Fisher’s exact test for categorical variables. Statistically significant values are bolded (p < 0.05). Cold ischemic time and donor type apply only to transplanted patients. HLA-DR mismatch was not available for suspended patients. GN: glomerulonephritis; PCKD: polycystic kidney disease; FSGS: focal segmental glomerulosclerosis; CKD: chronic kidney disease; HLA-DR: human leukocyte antigen-DR; DBD: donation after brain death; DCD: donation after circulatory death

Characteristic	Transplanted (n = 62)	Suspended (n = 45)	P-value¹
Age, years (median, range)	48.5 (18–74)	64 (28–75)	<0.001²
Male sex, n (%)	41 (66.1)	26 (57.8)	0.38³
BMI, kg/m² (median, range)	27.0 (19–35.6)	27.7 (18.3–36.2)	0.54²
Primary kidney disease, n (%)			0.07³
Diabetes	10 (16.1)	10 (22.2)	
GN	6 (9.7)	8 (17.8)	
IgA nephropathy	9 (14.5)	3 (6.7)	
PCKD	8 (12.9)	7 (15.6)	
FSGS	9 (14.5)	1 (2.2)	
CKD (other)	8 (12.9)	10 (22.2)	
Reflux nephropathy	2 (3.2)	2 (4.4)	
Other	10 (16.1)	4 (8.9)	
HLA-DR mismatch, n (%)			–
0	3 (4.8)	–	
1	26 (41.9)	–	
2	31 (50.0)	–	
Unknown	2 (3.2)	–	
Donor type (transplanted only), n (%)			–
Living donor	12 (19.4)	–	
DBD	34 (54.8)	–	
DCD	16 (25.8)	–	
Cold ischemic time, min (median, range)	493 (74–772)	–	–
Dialysis modality, n (%)			<0.001³
Hemodialysis	–	31 (68.9)	
Peritoneal dialysis	–	8 (17.8)	
Pre-emptive	–	6 (13.3)	

MeNTS scores and case selection

**Figure 1:**
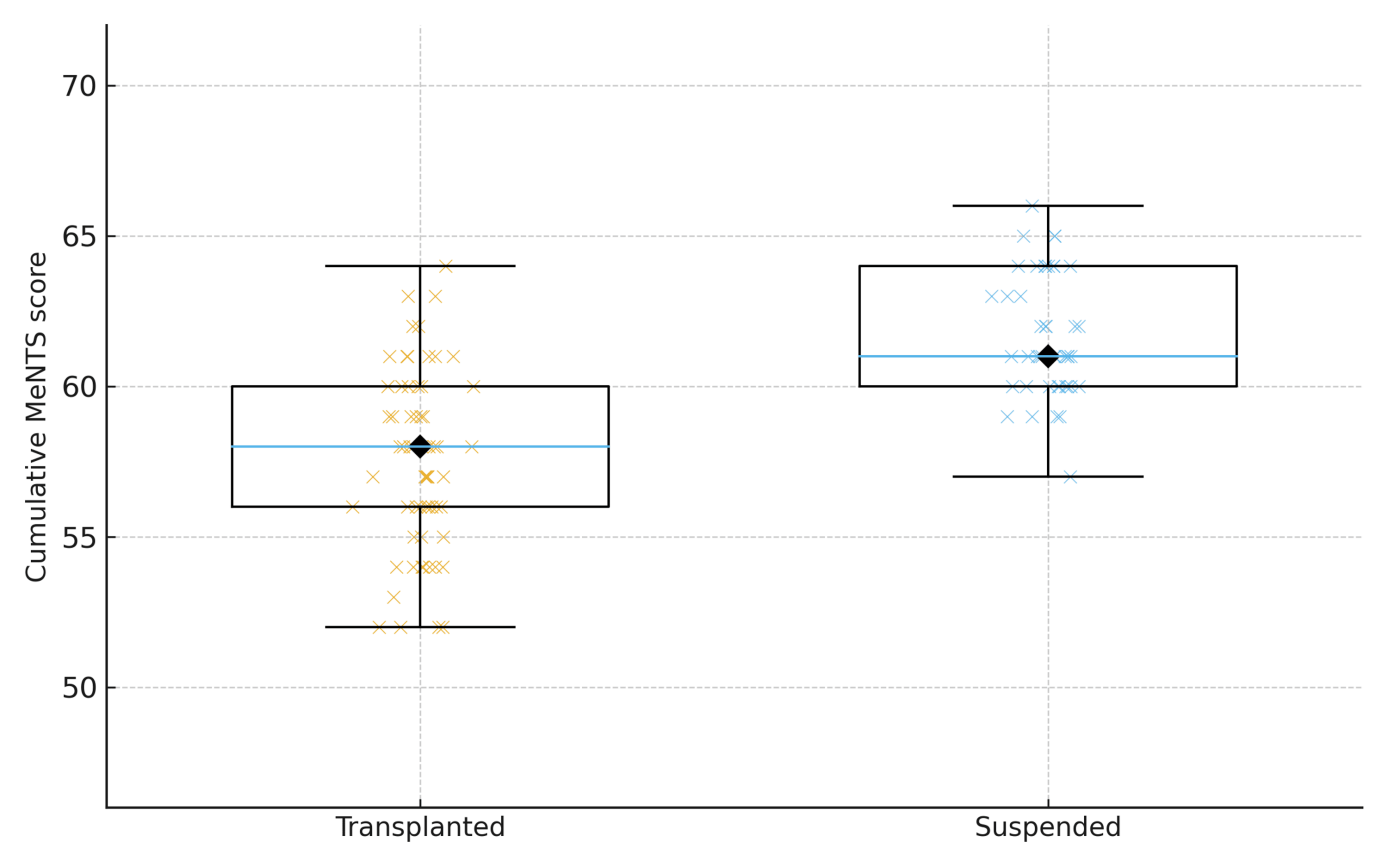
Distribution of cumulative MeNTS scores by selection status (Transplanted vs. Suspended). Boxplot of cumulative MeNTS scores for patients who proceeded to transplantation (Transplanted) and those whose procedures were suspended (Suspended). Individual points represent patient-level scores (jitter added for clarity); diamonds mark medians. Groups were compared using the Mann-Whitney U test (U = 401, p = 3.6 × 10^-10^), indicating significantly lower MeNTS scores among transplanted patients. Clinically, lower MeNTS scores were associated with selection for transplantation, consistent with prioritisation of patients judged to have lower peri-operative and transmission risk. MeNTS: Medically Necessary, Time-Sensitive

Cumulative MeNTS scores differed between groups: transplanted patients had a median score of 58 (range = 52-64; mean = 57.5) versus 61 (range = 57-65; mean = 61.6) in suspended patients. The difference in distributions was statistically significant (Mann-Whitney U = 401, Z = −6.269, p = 3.6 × 10⁻¹⁰), indicating selection of lower-MeNTS patients for transplantation during the study period (Figure 1).

Early graft and patient outcomes

One-year patient survival was high in both groups: 96.8% (60/62; 95% CI = 89.0-99.1%) in the transplanted group and 97.8% (44/45; 95% CI = 88.4-99.6%) in the suspended group. Among transplanted patients, DGF, defined as the requirement for dialysis within the first seven days post-transplant, occurred in 20/62 patients (32.3%; 95% CI = 22.0-44.6%). Five transplanted patients (5/62; 8.1%; 95% CI = 3.5-17.5%) subsequently developed graft failure during available follow-up. The median three-month eGFR among transplanted patients was 53 mL/minute/1.73 m² (range = 16-90). The median length of index hospital stay was eight days (range = 4-68). (DGF, graft failure, and eGFR are outcomes for the transplanted group only and therefore are reported descriptively.)

COVID-19 infections and severity

In total, 14 patients (13.1%; 95% CI = 8.0-20.8%) acquired laboratory-confirmed COVID-19 infection during follow-up: 9/62 (14.5%; 95% CI = 7.8-25.3%) in transplanted patients and 5/45 (11.1%; 95% CI = 4.8-23.5%) in suspended patients. Of these 14 infections, nine required hospital admission, three required intensive care, and one resulted in death. The difference in infection incidence between groups was not statistically significant (χ² = 0.26, p = 0.61).

Associations between MeNTS and outcomes

Higher MeNTS scores were strongly associated with selection for suspension rather than transplantation. In the transplanted subgroup, cumulative MeNTS score was not a significant predictor of early graft failure, 90‑day survival, or COVID-19 infection in univariate analyses (all p > 0.05), although analysis was limited by small event counts.

## Discussion

Principal findings

In this retrospective, single-center, cohort study of 107 patients considered for kidney transplantation during the first year of the COVID-19 pandemic, patients selected to proceed with transplantation had significantly lower MeNTS scores than those whose procedures were suspended. Despite the constrained environment, short-term outcomes were reassuring: one-year patient survival exceeded 96% in both groups, early graft function (median three-month eGFR of 53 mL/minute/1.73 m²) was acceptable, and graft failure occurred in 8.1% of transplanted patients. COVID-19 infection occurred in 13% of the cohort during follow-up, with a small number of severe cases and one COVID-19-related death. These findings suggest that, when used as part of a broader clinical strategy, MeNTS-informed selection (or clinical decision-making that mirrored MeNTS constructs) could identify patients with lower perioperative/transmission risk and permit safe continuation of transplantation in selected patients during the acute crisis.

Comparison with other studies

Our observation of reduced transplant activity and selective prioritization mirrors international reports from the early pandemic showing substantial declines in transplant volumes and redeployment of resources; kidney and living-donor programmes were among the most affected [8]. National datasets from the United Kingdom similarly documented a marked, but partial, reduction in activity in 2020/2021, followed by a gradual recovery. Taken together, these system-level data contextualize single-center experiences and highlight the value of explicit prioritization frameworks during capacity shocks.

Published evaluations of MeNTS and related triage instruments have shown that the tools can improve transparency and reproducibility of scheduling decisions and possess reasonable reliability in some specialty adaptations; however, studies have noted limitations in external validity and the need for procedure-specific recalibration [9]. In surgical subspecialties where MeNTS has been adapted, evidence supports its pragmatic utility but also emphasizes the importance of local validation [10]. Our findings align with these broader assessments: MeNTS appears useful as a rapid triage aid but requires transplant-specific consideration of long-term benefit and waiting-list harms.

Interpretation and implications

There are three implications from our data. First, structured prioritization, whether via MeNTS or an equivalent clinical algorithm, appears to have supported safe continuation of time-sensitive transplantation for patients with lower predicted perioperative and transmission risk. Second, the transplant context introduces unique trade-offs that general surgical triage instruments do not explicitly capture (e.g., the long-term survival benefit of transplantation, immunological complexity, and potential loss of organ offers), and these must be weighted in any transplant-specific adaptation. Third, the rapid evolution of COVID-19 countermeasures after 2020 (mass vaccination, booster dosing, tixagevimab/cilgavimab pre-exposure prophylaxis in selected immunocompromised patients, and oral antivirals such as nirmatrelvir-ritonavir for early treatment) materially changes the absolute perioperative risk for transplant candidates and recipients and therefore should be incorporated into prioritization decisions and future versions of any scoring system [11].

Strengths and limitations

This study’s strengths include consecutive case ascertainment across a complete pandemic year, transparent application of a widely discussed triage tool, and reporting of clinically meaningful outcomes (early graft function, DGF, graft failure, and COVID-19 severity). Nevertheless, important limitations temper interpretation. The single-center, retrospective design limits generalizability and introduces potential information bias in retrospective MeNTS scoring. Event counts for rare but critical outcomes (COVID-19-related mortality, ICU admission, graft failure) were small, limiting statistical power to detect modest associations. As our study also reflects the early pandemic context, before widespread booster programs and routine availability of some therapeutics, absolute risks reported here are not directly transferable to later phases of the pandemic [12].

## Conclusions

In this single-center, retrospective, cohort study of patients considered for kidney transplantation during the first year of the COVID-19 pandemic, selection for transplantation favored patients with lower MeNTS scores and was associated with acceptable short-term patient and graft outcomes. Early graft function, rates of DGF, and one-year survival observed in the transplanted cohort suggest that, when used alongside clinical judgement and local capacity assessment, structured prioritization can enable the safe continuation of time-sensitive transplant activity during acute system shocks. However, MeNTS in its original form does not capture several transplant-specific considerations that materially affect decision-making: notably, long-term survival benefit, immunological complexity, harms of prolonged dialysis, and the implications of declining organ offers. Since 2020, the risk landscape has shifted with widespread vaccination, booster programs, and targeted therapeutics; contemporary prioritization algorithms should therefore be prospectively validated and adapted for transplant settings, incorporate vaccination status and objective vaccine response measures where available, and be linked to prospective data collection and regional activity dashboards. Multi-center validation and registry-based analyses are needed to confirm generalizability, refine the weighting of prioritization items, and assess longer-term outcomes to ensure that transplant services are resilient in future crises.
